# Educational outcomes of a clinical and translational summer research internship program between a private university and a medical school

**DOI:** 10.21203/rs.3.rs-10677693/v1

**Published:** 2026-08-13

**Authors:** Matthew R. Allen, Melanie E. DeFord

**Affiliations:** Indiana University School of Medicine; University of Notre Dame

**Keywords:** workforce development, CTSA, summer program, immersion experience

## Abstract

Undergraduate institutions can have limited opportunities for learners to engage in clinical and translational research (CTR). In 2009, the Indiana Clinical and Translational Science Institute launched a summer research program to provide undergraduates from the University of Notre Dame with CTR experiences at the Indiana University School of Medicine. Scholars engage in full-time, 8-week summer research experiences. A total of 58 Scholars have participated. 27 went on to earn a medical degree (MD or DO), while others have pursued advanced degrees (PhD, JD, MBA, and MS). This private university–medical school partnership provides a model to support the CTR workforce.

## Background

The US has a persistent shortage of physician-scientists^1^. The underlying cause of this shortage is multifactorial, and approaches to overcome it are numerous. Engaging learners in research experiences has beneficial effects in shaping career trajectories^2–5^. There have been major advances in promoting research experiences to medical students. These help increase research literacy and can spark interest in, and open doors for, a physician-scientist career. Literature supports the benefits of immersion research experiences earlier in an educational career^6^. This is based on the idea that career plans can often be determined during undergraduate years (or earlier).

One challenge for many undergraduate institutions is providing clinical and translational research experiences for students with physician-scientist aspirations, which are important for reaching career goals and enhancing medical school applications. This is even more challenging for universities without direct affiliations with medical schools. The Indiana Clinical and Translational Science Institute (CTSI) is a statewide consortium focused on advancing health through research across the state and beyond. The Indiana CTSI includes the two largest R1 state universities (Indiana University and Purdue University) and the University of Notre Dame, a private R1 institution. In 2009, a program was launched to provide University of Notre Dame students with summer immersion experiences in clinical/translational research at the Indiana University School of Medicine, the only allopathic medical school in the state and one of the largest in the country. This short communication describes our program and the educational outcomes (terminal degrees) of participants between 2009 and 2025.

## Activity

The Indiana CTSI Summer undergraduate program is designed to connect students from the University of Notre Dame with research teams at the Indiana University School of Medicine (IUSM). The selected students (termed Scholars) spend a summer (8 weeks) working on research projects. The goal is to increase their awareness of, and interest in, translational research designed to accelerate the pace of moving therapies from the “bench to bedside.” The program generally runs from June through July each summer, and students are expected to engage in research and related activities full time (40 hours per week). Students are supported with a research stipend ($4,000 in 2026). If students live > 40 miles from downtown Indianapolis (the location of the main IUSM research campus), campus housing is provided.

Any undergraduate student at the University of Notre Dame who has completed at least one full year of undergraduate education and is enrolled full-time in an undergraduate degree program in a health or life science discipline is eligible to apply. Only US citizens or permanent residents are eligible. In addition to basic demographic information, applicants are asked about their academic aspirations, research experiences, extracurricular activities, awards/academic achievements, and past jobs. Applicants are asked to upload an academic transcript (does not need to be official), and one letter of reference. A personal statement and CV/resume are both optional. Applicants are evaluated by faculty and graduate students at the IU School of Medicine using a rubric with a 1–3 scale across 5 areas (Fig. 1). Each applicant is evaluated by multiple reviewers. Since the inception of the program, up to four Scholars have been selected each year.

Scholars engage in safety and ethics training at the outset of the program, and in several professional development programs throughout the 8-week period. Scholars working with physician mentors are encouraged to engage in clinical shadowing; those matched with non-physician mentors are connected with alternative faculty for shadowing opportunities. The Scholars present a research poster at a local symposium at the conclusion of the program.

Terminal educational degrees, the primary outcome of this evaluation, of Scholars from 2009 through 2025 were collected using three methods: 1) the National Student Clearinghouse, a non-profit database of higher education degrees; 2) LinkedIn, an online professional network; 3) University databases (University of Notre Dame and Indiana University). As this work was done for the original purpose of program quality improvement, we did not obtain informed consent from the students in the program. This work/study was approved by the Indiana University IRB (Exempt status – protocol 31148) in accordance with federal regulation (45 CFR 46) and institutional guidelines.

## Results

A total of 58 Scholars from the University of Notre Dame have engaged in the program since 2009 (Fig. 2A). At least two Scholars participated each year (except 2020 during COVID), with a maximum of four Scholars in any given year. All Scholars have either graduated with a bachelor’s degree (51) or are still enrolled (7) at the University of Notre Dame.

A total of 27 Scholars went on to earn a medical degree (MD or DO). Another 3 are currently enrolled in medical school (Fig. 2B). Five Scholars earned PhDs, two earned a JD, 1 Scholar earned a Master of Business Administration (MBA), and 3 earned terminal Master of Science degrees (MS). There were 10 Scholars (20%) for which post-undergraduate data were not available.

## Discussion

The Clinical and Translational Science award program, under the leadership of the National Center for Advancing Clinical Science (NCATS), was designed to support networks that can drive research discoveries and improve care. Among the goals of the CTSA program are to promote regional partnerships and to create clinical and translational science training and career support programs. The Indiana CTSI, from its inception, has invested in programs that advance the workforce. The Indiana CTSI Summer undergraduate program capitalizes on the unique partnership between the University of Notre Dame and the Indiana University School of Medicine – providing undergraduates an immersive experience in clinical and translational research. As shown by our outcome data, over 50% of the students who have engaged in the program have gone on to medical school – a key step toward a career as a physician-scientist. Additional Scholars have pursued advanced degrees.

Private undergraduate universities offer several strengths in their academic curricula, and many have robust research programs. Although possible, private universities may have limited opportunities for students to work in research environments with a clinical/translational focus. Our partnership program, between a private university and a medical school/academic health center, provides an approach to offer students a clinical/translational research experience. The program requires minimal, yet important infrastructure, including funding for stipends/housing and someone to oversee/manage the overall program. In our program, the stipend and housing costs are supported by the University of Notre Dame while the program leadership is supported through the Indiana CTSI. Mentors/research teams who are open to hosting undergraduates into their environments are also foundational to a successful program. IUSM has a long-standing commitment to valuing mentorship of trainees, which contributed to this program’s launch and longevity.

In conclusion, this private university/medical school partnership has allowed Scholars to engage with clinical and translational research teams for a summer research experience. Most Scholars went on to earn medical or other advanced degrees. This program can serve as a model for undergraduate institutions to build novel medical research experiences for their students through academic partnerships.

## Figures and Tables

**Figure 1:**
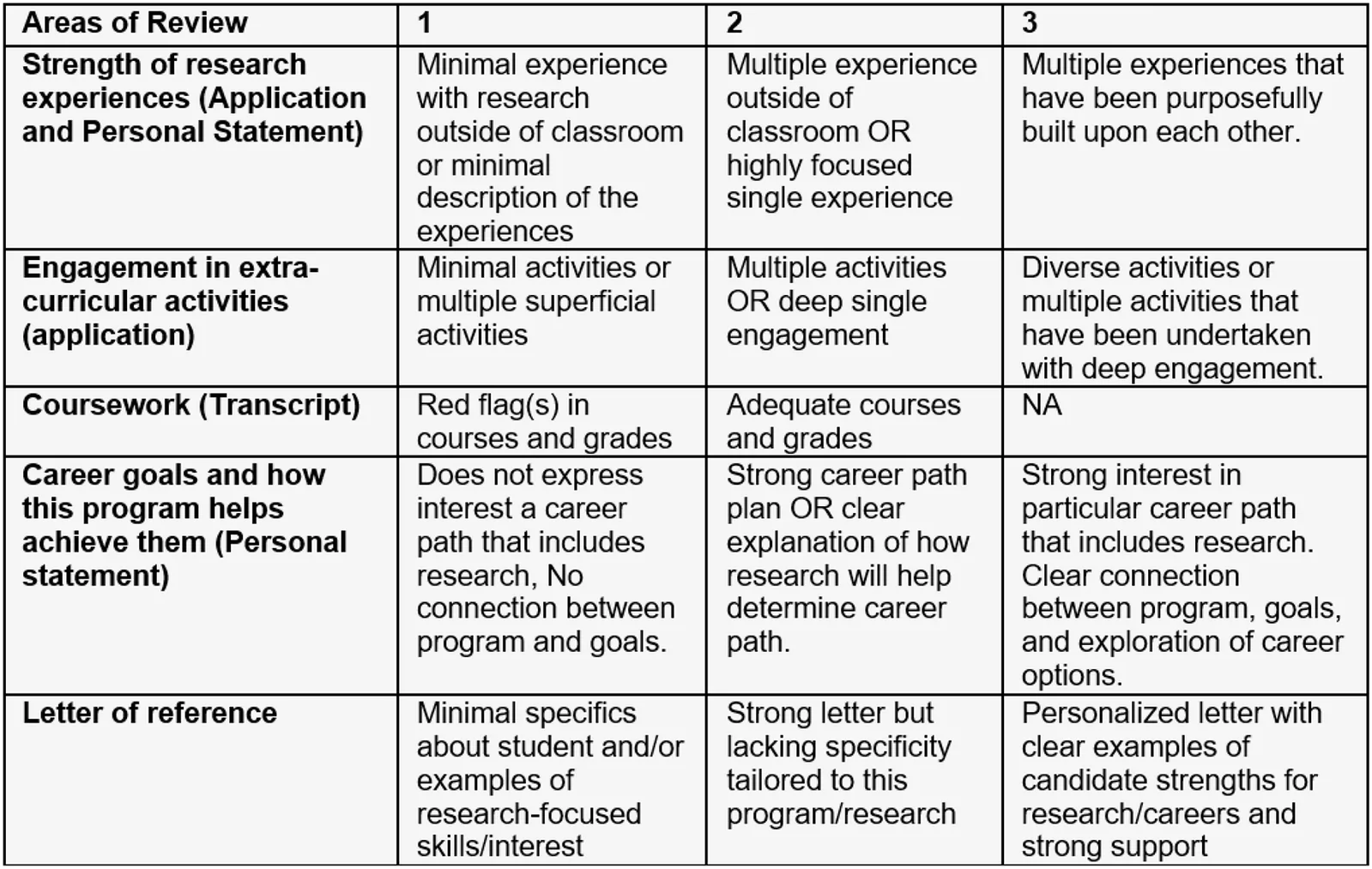
Evaluation rubric for summer program application

**Figure 2:**
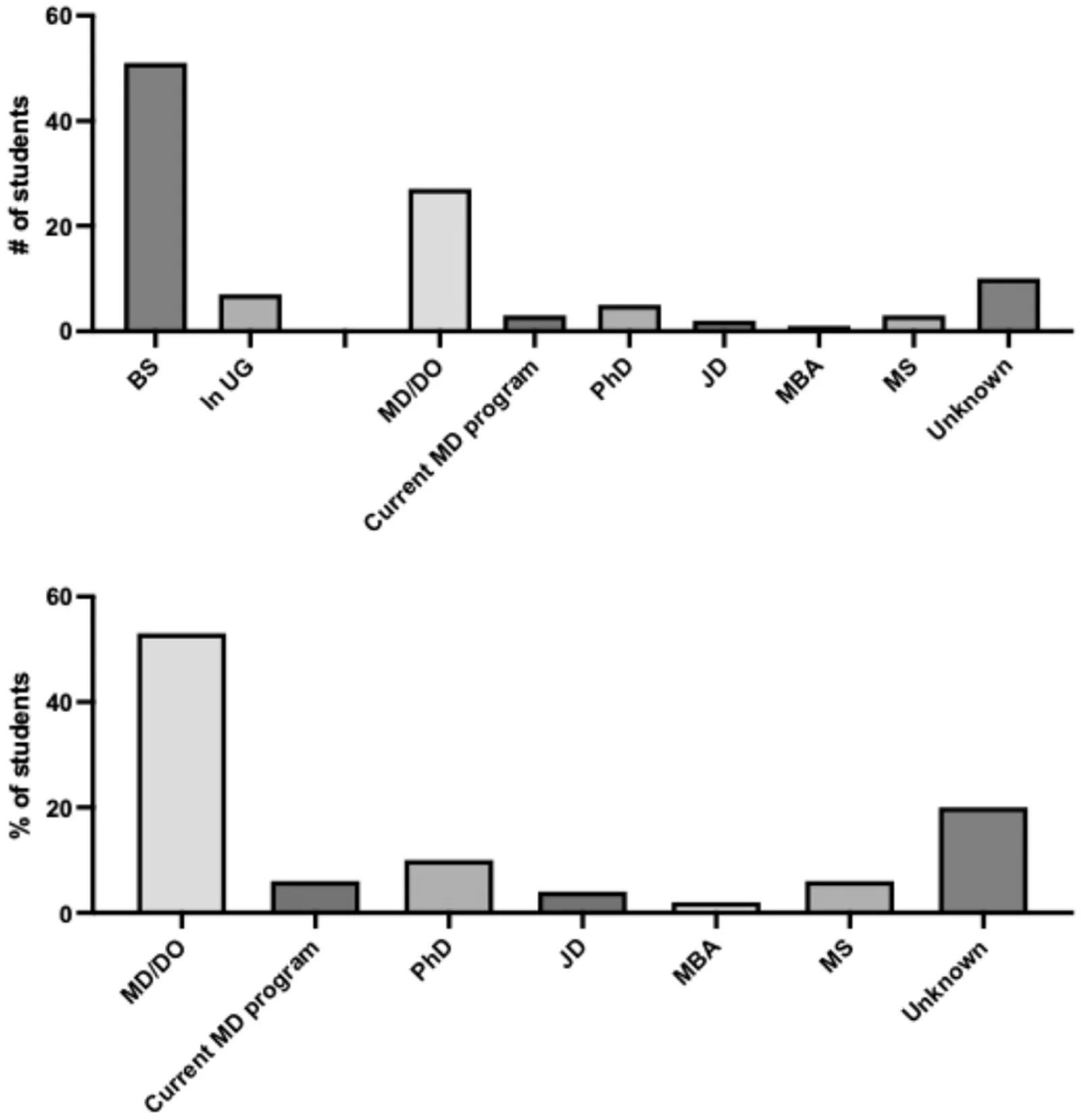
Absolute (top) and percentage (bottom) of terminal degrees (or current degree programs) from program participants.

## Data Availability

All data generated or analysed during this study are included in this published article
